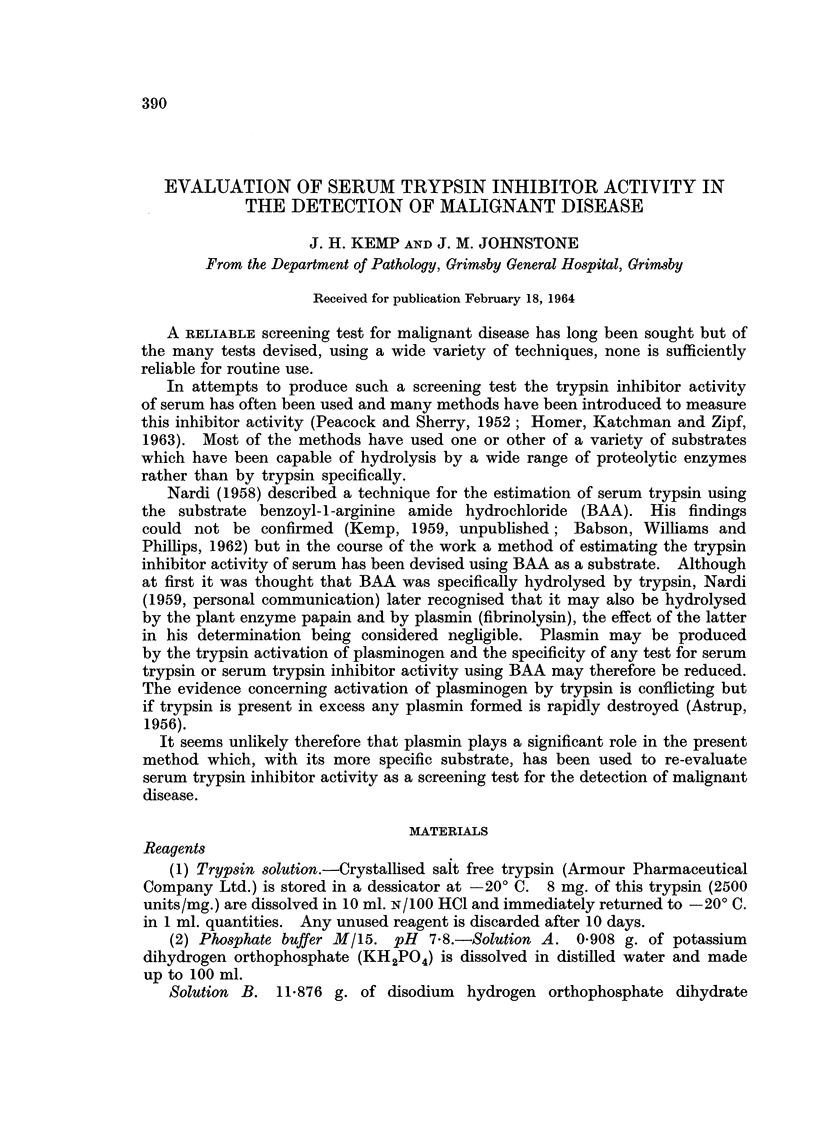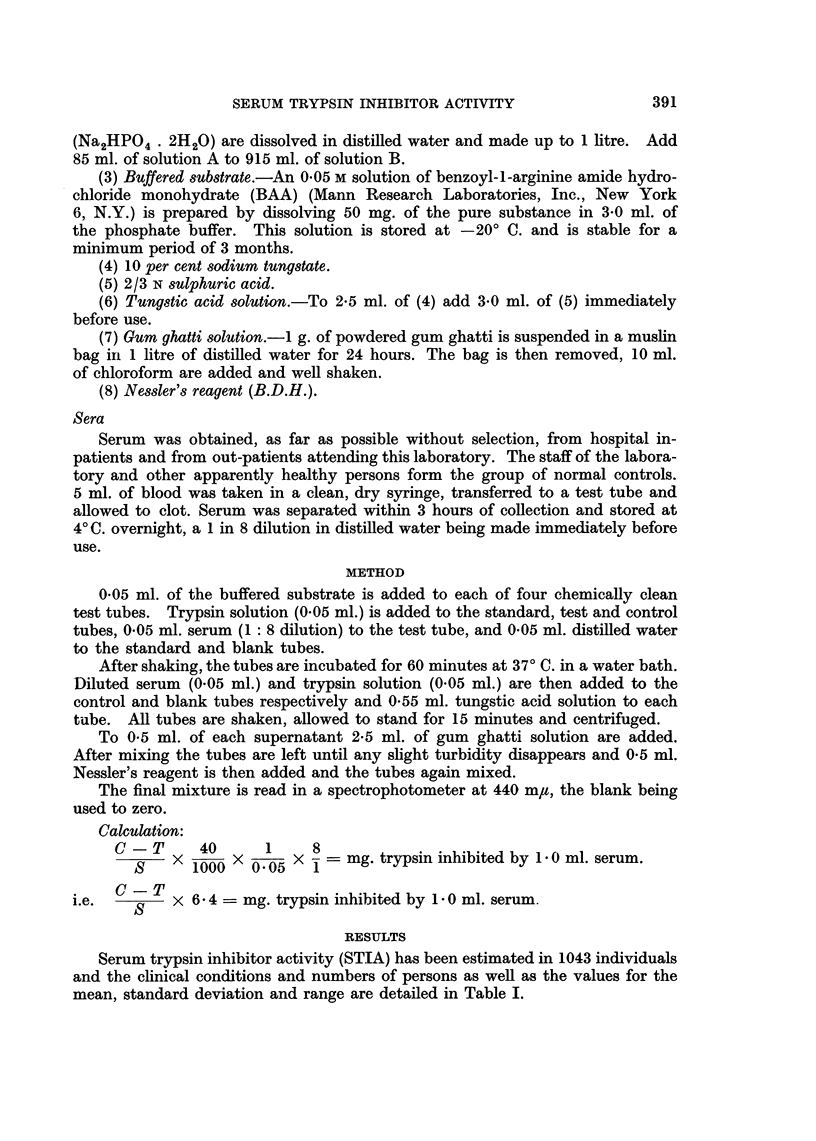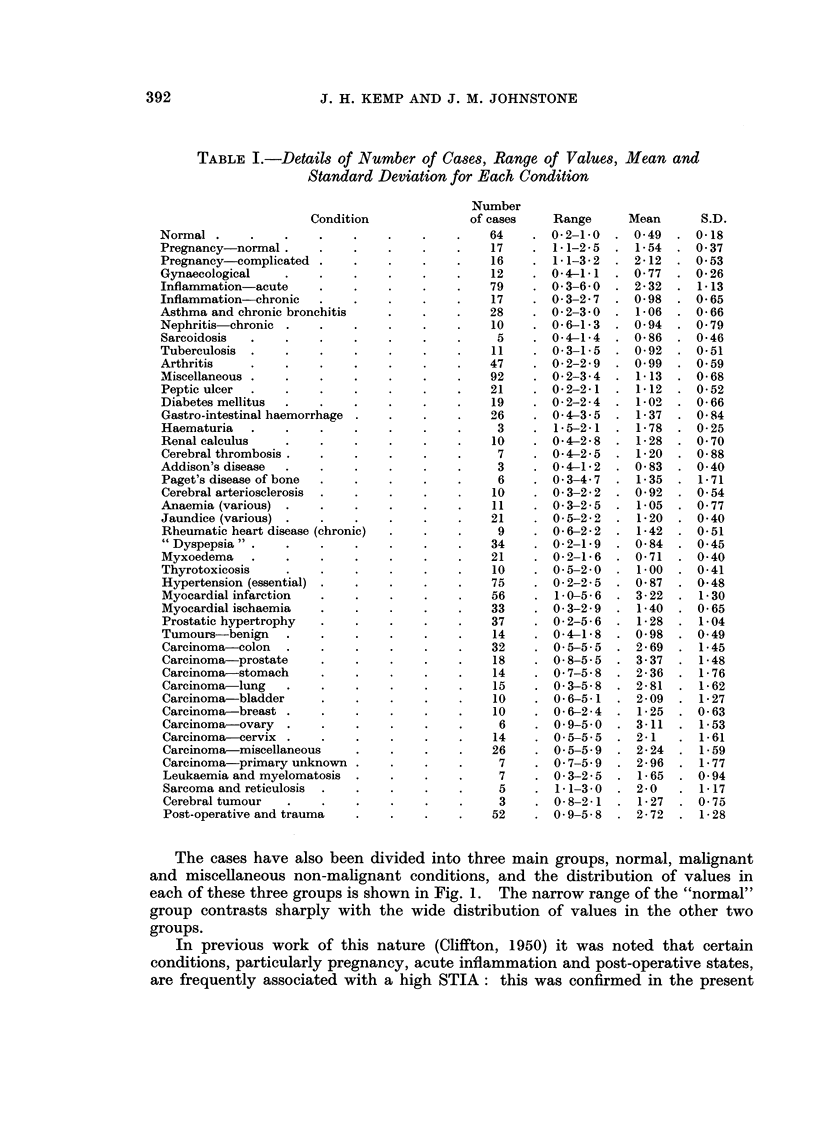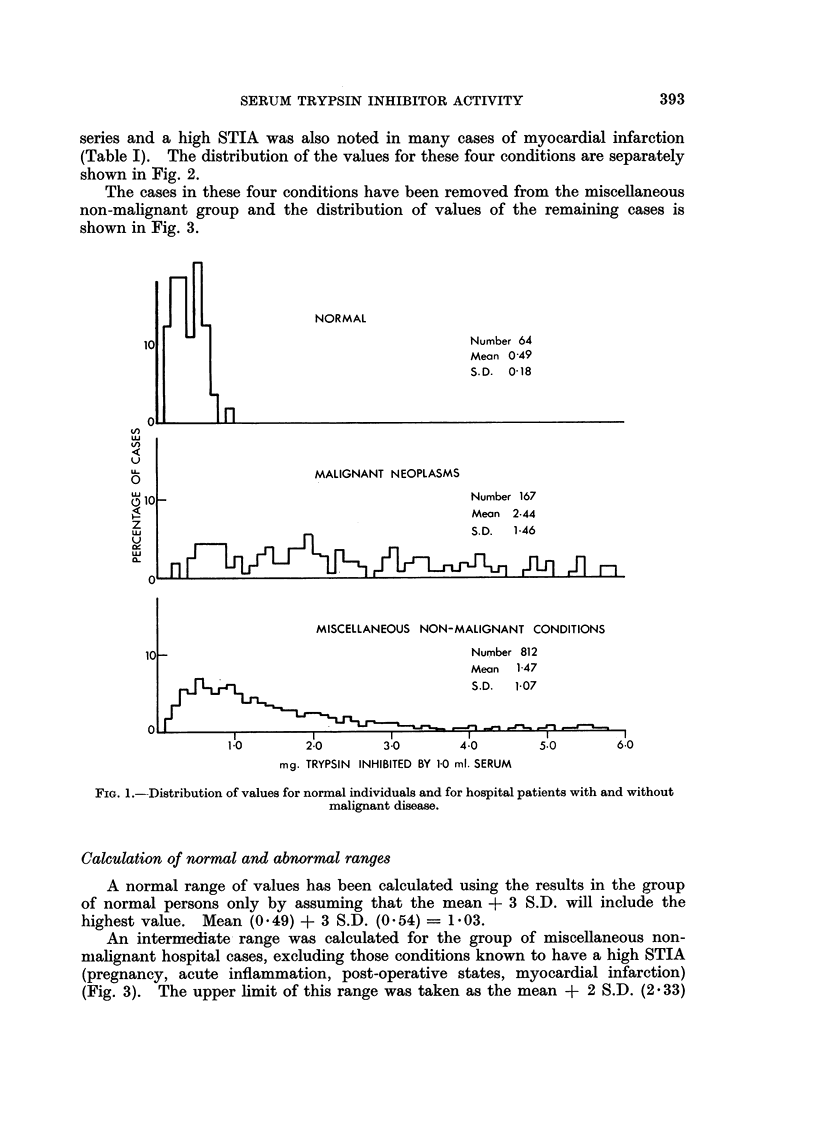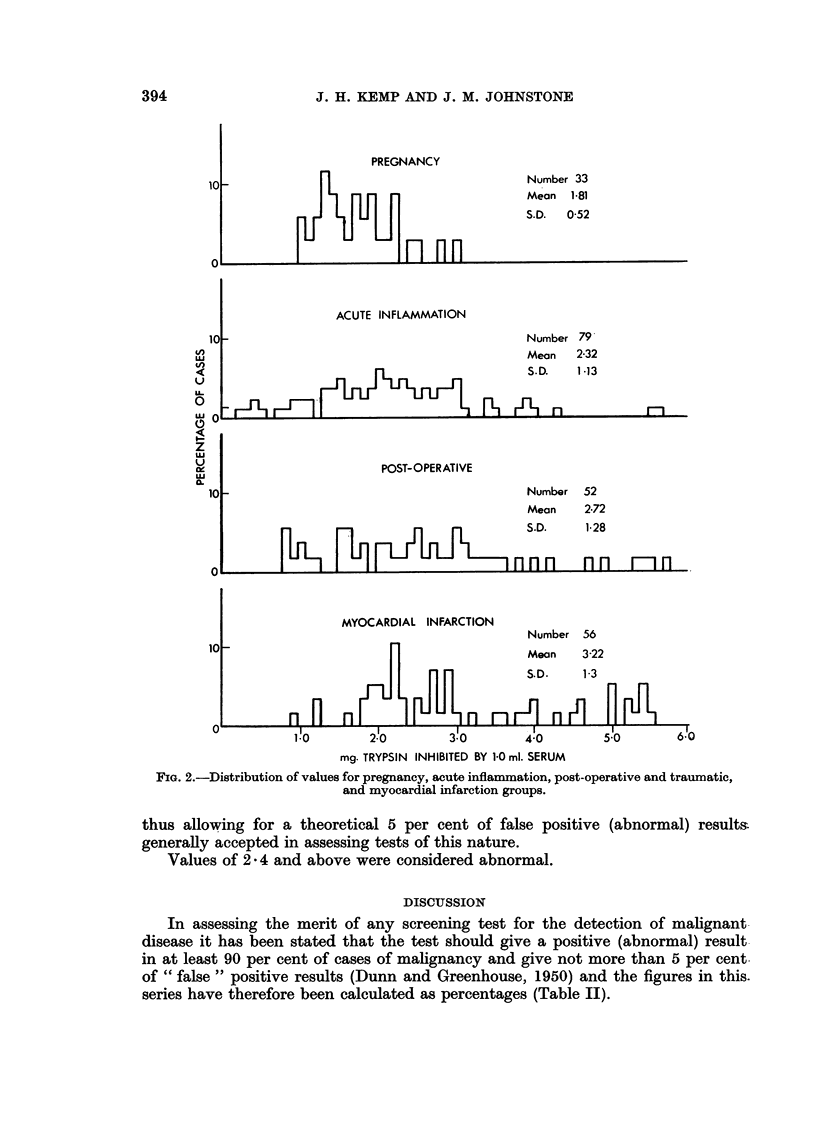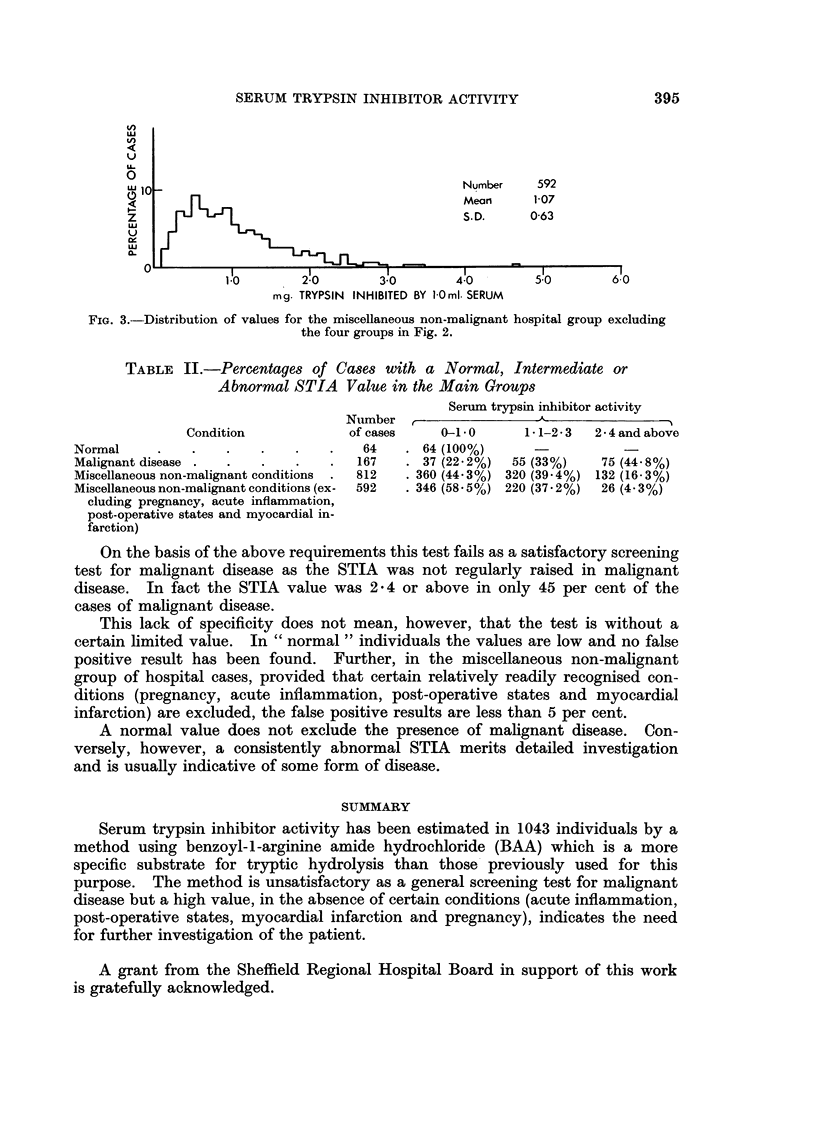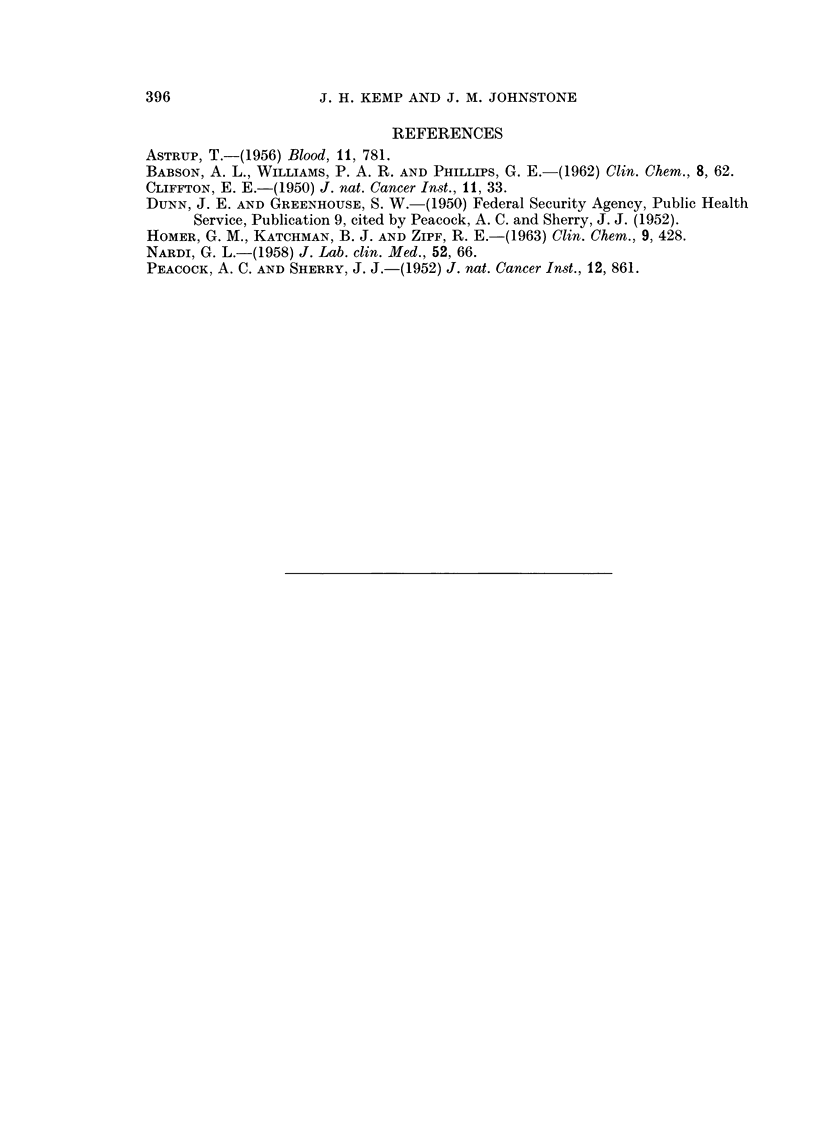# Evaluation of Serum Trypsin Inhibitor Activity in the Detection of Malignant Disease

**DOI:** 10.1038/bjc.1964.45

**Published:** 1964-06

**Authors:** J. H. Kemp, J. M. Johnstone


### EVALUATION OF SERUM TRYPSIN INHIBITOR ACTIVITY IN THE DETECTION OF MALIGNANT DISEASE

J. H. KEMP AND J. M. JOHNSTONE

*From the Department of Pathology, Grimsby General Hospital, Grimsby*

Received for publication February 18, 1964

A RELIABLE screening test for malignant disease has long been sought but of the many tests devised, using a wide variety of techniques, none is sufficiently reliable for routine use.

In attempts to produce such a screening test the trypsin inhibitor activity of serum has often been used and many methods have been introduced to measure this inhibitor activity (Peacock and Sherry, 1952 ; Homer, Katchman and Zipf, 1963). Most of the methods have used one or other of a variety of substrates which have been capable of hydrolysis by a wide range of proteolytic enzymes rather than by trypsin specifically.

Nardi (1958) described a technique for the estimation of serum trypsin using the substrate benzoyl-1-arginine amide hydrochloride (BAA). His findings could not be confirmed (Kemp, 1959, unpublished ; Babson, Williams and Phillips, 1962) but in the course of the work a method of estimating the trypsin inhibitor activity of serum has been devised using BAA as a substrate. Although at first it was thought that BAA was specifically hydrolysed by trypsin, Nardi (1959, personal communication) later recognised that it may also be hydrolysed by the plant enzyme papain and by plasmin (fibrinolysin), the effect of the latter in his determination being considered negligible. Plasmin may be produced by the trypsin activation of plasminogen and the specificity of any test for serum trypsin or serum trypsin inhibitor activity using BAA may therefore be reduced. The evidence concerning activation of plasminogen by trypsin is conflicting but if trypsin is present in excess any plasmin formed is rapidly destroyed (Astrup, 1956).

It seems unlikely therefore that plasmin plays a significant role in the present method which, with its more specific substrate, has been used to re-evaluate serum trypsin inhibitor activity as a screening test for the detection of malignant disease.

### MATERIALS

### *Reagents*

(1) *Trypsin solution.*—Crystallised salt free trypsin (Armour Pharmaceutical Company Ltd.) is stored in a dessicator at —20° C. 8 mg. of this trypsin (2500 units/mg.) are dissolved in 10 ml. N/100 HCl and immediately returned to —20° C. in 1 ml. quantities. Any unused reagent is discarded after 10 days.

(2) *Phosphate buffer M/15. pH* 7·8.—*Solution A.* 0·908 g. of potassium dihydrogen orthophosphate ($KH_2PO_4$) is dissolved in distilled water and made up to 100 ml.

*Solution B.* 11·876 g. of disodium hydrogen orthophosphate dihydrate

($Na_2HPO_4 . 2H_2O$) are dissolved in distilled water and made up to 1 litre. Add 85 ml. of solution A to 915 ml. of solution B.

(3) *Buffered substrate.*—An 0·05 M solution of benzoyl-1-arginine amide hydrochloride monohydrate (BAA) (Mann Research Laboratories, Inc., New York 6, N.Y.) is prepared by dissolving 50 mg. of the pure substance in 3·0 ml. of the phosphate buffer. This solution is stored at −20° C. and is stable for a minimum period of 3 months.

(4) 10 *per cent sodium tungstate.*

(5) 2/3 N *sulphuric acid.*

(6) *Tungstic acid solution.*—To 2·5 ml. of (4) add 3·0 ml. of (5) immediately before use.

(7) *Gum ghatti solution.*—1 g. of powdered gum ghatti is suspended in a muslin bag in 1 litre of distilled water for 24 hours. The bag is then removed, 10 ml. of chloroform are added and well shaken.

(8) *Nessler's reagent* (*B.D.H.*).

*Sera*

Serum was obtained, as far as possible without selection, from hospital in-patients and from out-patients attending this laboratory. The staff of the laboratory and other apparently healthy persons form the group of normal controls. 5 ml. of blood was taken in a clean, dry syringe, transferred to a test tube and allowed to clot. Serum was separated within 3 hours of collection and stored at 4° C. overnight, a 1 in 8 dilution in distilled water being made immediately before use.

### METHOD

0·05 ml. of the buffered substrate is added to each of four chemically clean test tubes. Trypsin solution (0·05 ml.) is added to the standard, test and control tubes, 0·05 ml. serum (1 : 8 dilution) to the test tube, and 0·05 ml. distilled water to the standard and blank tubes.

After shaking, the tubes are incubated for 60 minutes at 37° C. in a water bath. Diluted serum (0·05 ml.) and trypsin solution (0·05 ml.) are then added to the control and blank tubes respectively and 0·55 ml. tungstic acid solution to each tube. All tubes are shaken, allowed to stand for 15 minutes and centrifuged.

To 0·5 ml. of each supernatant 2·5 ml. of gum ghatti solution are added. After mixing the tubes are left until any slight turbidity disappears and 0·5 ml. Nessler's reagent is then added and the tubes again mixed.

The final mixture is read in a spectrophotometer at 440 m$\mu$, the blank being used to zero.

*Calculation*:

$$\frac{C-T}{S} \times \frac{40}{1000} \times \frac{1}{0\cdot 05} \times \frac{8}{1} = \text{mg. trypsin inhibited by } 1\cdot 0 \text{ ml. serum.}$$

i.e. $$\frac{C-T}{S} \times 6\cdot 4 = \text{mg. trypsin inhibited by } 1\cdot 0 \text{ ml. serum.}$$

### RESULTS

Serum trypsin inhibitor activity (STIA) has been estimated in 1043 individuals and the clinical conditions and numbers of persons as well as the values for the mean, standard deviation and range are detailed in Table I.

TABLE I.—*Details of Number of Cases, Range of Values, Mean and Standard Deviation for Each Condition*

| Condition | Number of cases | Range | Mean | S.D. |
|---|---|---|---|---|
| Normal | 64 | 0·2–1·0 | 0·49 | 0·18 |
| Pregnancy—normal | 17 | 1·1–2·5 | 1·54 | 0·37 |
| Pregnancy—complicated | 16 | 1·1–3·2 | 2·12 | 0·53 |
| Gynaecological | 12 | 0·4–1·1 | 0·77 | 0·26 |
| Inflammation—acute | 79 | 0·3–6·0 | 2·32 | 1·13 |
| Inflammation—chronic | 17 | 0·3–2·7 | 0·98 | 0·65 |
| Asthma and chronic bronchitis | 28 | 0·2–3·0 | 1·06 | 0·66 |
| Nephritis—chronic | 10 | 0·6–1·3 | 0·94 | 0·79 |
| Sarcoidosis | 5 | 0·4–1·4 | 0·86 | 0·46 |
| Tuberculosis | 11 | 0·3–1·5 | 0·92 | 0·51 |
| Arthritis | 47 | 0·2–2·9 | 0·99 | 0·59 |
| Miscellaneous | 92 | 0·2–3·4 | 1·13 | 0·68 |
| Peptic ulcer | 21 | 0·2–2·1 | 1·12 | 0·52 |
| Diabetes mellitus | 19 | 0·2–2·4 | 1·02 | 0·66 |
| Gastro-intestinal haemorrhage | 26 | 0·4–3·5 | 1·37 | 0·84 |
| Haematuria | 3 | 1·5–2·1 | 1·78 | 0·25 |
| Renal calculus | 10 | 0·4–2·8 | 1·28 | 0·70 |
| Cerebral thrombosis | 7 | 0·4–2·5 | 1·20 | 0·88 |
| Addison's disease | 3 | 0·4–1·2 | 0·83 | 0·40 |
| Paget's disease of bone | 6 | 0·3–4·7 | 1·35 | 1·71 |
| Cerebral arteriosclerosis | 10 | 0·3–2·2 | 0·92 | 0·54 |
| Anaemia (various) | 11 | 0·3–2·5 | 1·05 | 0·77 |
| Jaundice (various) | 21 | 0·5–2·2 | 1·20 | 0·40 |
| Rheumatic heart disease (chronic) | 9 | 0·6–2·2 | 1·42 | 0·51 |
| " Dyspepsia " | 34 | 0·2–1·9 | 0·84 | 0·45 |
| Myxoedema | 21 | 0·2–1·6 | 0·71 | 0·40 |
| Thyrotoxicosis | 10 | 0·5–2·0 | 1·00 | 0·41 |
| Hypertension (essential) | 75 | 0·2–2·5 | 0·87 | 0·48 |
| Myocardial infarction | 56 | 1·0–5·6 | 3·22 | 1·30 |
| Myocardial ischaemia | 33 | 0·3–2·9 | 1·40 | 0·65 |
| Prostatic hypertrophy | 37 | 0·2–5·6 | 1·28 | 1·04 |
| Tumours—benign | 14 | 0·4–1·8 | 0·98 | 0·49 |
| Carcinoma—colon | 32 | 0·5–5·5 | 2·69 | 1·45 |
| Carcinoma—prostate | 18 | 0·8–5·5 | 3·37 | 1·48 |
| Carcinoma—stomach | 14 | 0·7–5·8 | 2·36 | 1·76 |
| Carcinoma—lung | 15 | 0·3–5·8 | 2·81 | 1·62 |
| Carcinoma—bladder | 10 | 0·6–5·1 | 2·09 | 1·27 |
| Carcinoma—breast | 10 | 0·6–2·4 | 1·25 | 0·63 |
| Carcinoma—ovary | 6 | 0·9–5·0 | 3·11 | 1·53 |
| Carcinoma—cervix | 14 | 0·5–5·5 | 2·1 | 1·61 |
| Carcinoma—miscellaneous | 26 | 0·5–5·9 | 2·24 | 1·59 |
| Carcinoma—primary unknown | 7 | 0·7–5·9 | 2·96 | 1·77 |
| Leukaemia and myelomatosis | 7 | 0·3–2·5 | 1·65 | 0·94 |
| Sarcoma and reticulosis | 5 | 1·1–3·0 | 2·0 | 1·17 |
| Cerebral tumour | 3 | 0·8–2·1 | 1·27 | 0·75 |
| Post-operative and trauma | 52 | 0·9–5·8 | 2·72 | 1·28 |

The cases have also been divided into three main groups, normal, malignant and miscellaneous non-malignant conditions, and the distribution of values in each of these three groups is shown in Fig. 1. The narrow range of the "normal" group contrasts sharply with the wide distribution of values in the other two groups.

In previous work of this nature (Cliffton, 1950) it was noted that certain conditions, particularly pregnancy, acute inflammation and post-operative states, are frequently associated with a high STIA : this was confirmed in the present series and a high STIA was also noted in many cases of myocardial infarction (Table I). The distribution of the values for these four conditions are separately shown in Fig. 2.

The cases in these four conditions have been removed from the miscellaneous non-malignant group and the distribution of values of the remaining cases is shown in Fig. 3.

FIG. 1.—Distribution of values for normal individuals and for hospital patients with and without malignant disease.

*Calculation of normal and abnormal ranges*

A normal range of values has been calculated using the results in the group of normal persons only by assuming that the mean + 3 S.D. will include the highest value. Mean (0·49) + 3 S.D. (0·54) = 1·03.

An intermediate range was calculated for the group of miscellaneous non-malignant hospital cases, excluding those conditions known to have a high STIA (pregnancy, acute inflammation, post-operative states, myocardial infarction) (Fig. 3). The upper limit of this range was taken as the mean + 2 S.D. (2·33)

FIG. 2.—Distribution of values for pregnancy, acute inflammation, post-operative and traumatic, and myocardial infarction groups.

thus allowing for a theoretical 5 per cent of false positive (abnormal) results generally accepted in assessing tests of this nature.

Values of 2·4 and above were considered abnormal.

### DISCUSSION

In assessing the merit of any screening test for the detection of malignant disease it has been stated that the test should give a positive (abnormal) result in at least 90 per cent of cases of malignancy and give not more than 5 per cent of " false " positive results (Dunn and Greenhouse, 1950) and the figures in this series have therefore been calculated as percentages (Table II).

FIG. 3.—Distribution of values for the miscellaneous non-malignant hospital group excluding the four groups in Fig. 2.

TABLE II.—*Percentages of Cases with a Normal, Intermediate or Abnormal STIA Value in the Main Groups*

| Condition | Number of cases | Serum trypsin inhibitor activity 0–1·0 | 1·1–2·3 | 2·4 and above |
|---|---|---|---|---|
| Normal . . . . . . . | 64 | . 64 (100%) | — | — |
| Malignant disease . . . . . | 167 | . 37 (22·2%) | 55 (33%) | 75 (44·8%) |
| Miscellaneous non-malignant conditions . | 812 | . 360 (44·3%) | 320 (39·4%) | 132 (16·3%) |
| Miscellaneous non-malignant conditions (excluding pregnancy, acute inflammation, post-operative states and myocardial infarction) | 592 | . 346 (58·5%) | 220 (37·2%) | 26 (4·3%) |

On the basis of the above requirements this test fails as a satisfactory screening test for malignant disease as the STIA was not regularly raised in malignant disease. In fact the STIA value was 2·4 or above in only 45 per cent of the cases of malignant disease.

This lack of specificity does not mean, however, that the test is without a certain limited value. In " normal " individuals the values are low and no false positive result has been found. Further, in the miscellaneous non-malignant group of hospital cases, provided that certain relatively readily recognised conditions (pregnancy, acute inflammation, post-operative states and myocardial infarction) are excluded, the false positive results are less than 5 per cent.

A normal value does not exclude the presence of malignant disease. Conversely, however, a consistently abnormal STIA merits detailed investigation and is usually indicative of some form of disease.

### SUMMARY

Serum trypsin inhibitor activity has been estimated in 1043 individuals by a method using benzoyl-l-arginine amide hydrochloride (BAA) which is a more specific substrate for tryptic hydrolysis than those previously used for this purpose. The method is unsatisfactory as a general screening test for malignant disease but a high value, in the absence of certain conditions (acute inflammation, post-operative states, myocardial infarction and pregnancy), indicates the need for further investigation of the patient.

A grant from the Sheffield Regional Hospital Board in support of this work is gratefully acknowledged.

### REFERENCES

Astrup, T.—(1956) *Blood*, **11**, 781.
Babson, A. L., Williams, P. A. R. and Phillips, G. E.—(1962) *Clin. Chem.*, **8**, 62.
Cliffton, E. E.—(1950) *J. nat. Cancer Inst.*, **11**, 33.
Dunn, J. E. and Greenhouse, S. W.—(1950) Federal Security Agency, Public Health Service, Publication 9, cited by Peacock, A. C. and Sherry, J. J. (1952).
Homer, G. M., Katchman, B. J. and Zipf, R. E.—(1963) *Clin. Chem.*, **9**, 428.
Nardi, G. L.—(1958) *J. Lab. clin. Med.*, **52**, 66.
Peacock, A. C. and Sherry, J. J.—(1952) *J. nat. Cancer Inst.*, **12**, 861.